# Efficacy of Varenicline in the Treatment of Alcohol Dependence: An Updated Meta-Analysis and Meta-Regression

**DOI:** 10.3390/ijerph20054091

**Published:** 2023-02-24

**Authors:** Wiraphol Phimarn, Rotjanawat Sakhancord, Peerasaran Paitoon, Kritsanee Saramunee, Bunleu Sungthong

**Affiliations:** 1 Social Pharmacy Research Unit, Faculty of Pharmacy, Mahasarakham University, Kantharawichai District, Maha Sarakham 44150, Thailand; 2 Integrative Pharmaceuticals and Innovative of Pharmaceutical Technology Research Unit, Faculty of Pharmacy, Mahasarakham University, Kantharawichai District, Maha Sarakham 44150, Thailand

**Keywords:** varenicline, alcohol dependence, alcohol abstinence, drinks, craving

## Abstract

Background: Although varenicline has been used for alcohol dependence (AD) treatment, its efficacy for this condition remains controversial. Aims: This systematic review and meta-analysis of randomized controlled trials (RCTs) assesses the efficacy and safety of varenicline in patients with AD. Methods: PubMed, Cochrane Library, ScienceDirect, Web of Science, and ThaiLis were systematically searched. RCTs investigating the efficacy and safety of varenicline in patients with AD were included. Study selection, data extraction, and quality assessment were independently performed by two authors. The Jadad score and Cochrane risk of bias were used to assess the quality of the included studies. Heterogeneity was assessed using I^2^ and chi-squared tests. Results: Twenty-two high-quality RCTs on 1421 participants were included. Varenicline significantly reduced alcohol-related outcomes compared with placebo based on percentage of abstinent days (standardized mean difference [SMD] 4.20 days; 95% confidence interval [CI]: 0.21, 8.19; *p* = 0.04), drinks per day (SMD −0.23 drinks; 95% CI: −0.43, −0.04; *p* = 0.02), drinks per drinking day (SMD −0.24 drinks; 95% CI: −0.44, −0.05; *p* = 0.01), craving assessed using the Penn alcohol craving scale (SMD −0.35; 95% CI: −0.59, −0.12; *p* = 0.003), and craving assessed using the alcohol urge questionnaire (SMD −1.41; 95% CI: −2.12, −0.71; *p* < 0.0001). However, there were no significant effects on abstinence rate, percentage of drinking days, percentage of heavy drinking days, alcohol intoxication, or drug compliance. Serious side effects were not observed in the varenicline or placebo groups. Conclusion: Our results indicated that AD patients treated with varenicline showed improvement in percentage of very heavy drinking days, percentage of abstinent days, drinks per day, drinks per drinking day, and craving. However, well-designed RCTs with a large sample size and long duration on varenicline treatment in AD remain warranted to confirm our findings.

## 1. Introduction

Excessive alcohol consumption remains a crucial public health concern that affects both the economy and society. There are approximately 3 million alcohol-related mortalities annually worldwide, accounting for 5.3% of all mortalities, and the mortality continues to increase, especially among those aged 20–39 years [1]. Moreover, alcohol consumption is associated with the occurrence of more than 200 diseases, including psychological disorders and non-communicable diseases, such as cirrhosis, cancer, and cardiovascular diseases [2]. Alcohol dependence (AD) refers to the pattern of continuous and increasing consumption of alcohol often associated with physical and psychological consequences [3]. In the International Classification of Diseases (ICD)-11, alcohol use disorder (AUD) is either diagnosed as “alcohol dependence” or a “harmful pattern of the use of alcohol”. Moreover, the ICD-11 expanded on a group of substances, such as alcohol, nicotine, caffeine, and cannabis. Alcohol use disorders are classified as substance use and addictive behaviors. The proposed ICD-11 classification sharply contradicts the fifth version of the Diagnostic and Statistical Manual of Mental Disorders (DSM-5). The proposed ICD-11 represents not only a simplification, but also an expansion of the ICD-10 dependence features (WHO terminology for criteria) [4] and includes some potentially significant changes, while the DSM-IV and ICD-10 share similar criteria for dependence [5].

Alcohol consumption can have some consequences, such as automotive accidents, quarrels, and assaults, as well as long-term effects, such as an alcohol addiction and financial difficulties [6,7]. AD can be divided into four stages, namely (1) pre-AD, (2) early AD, (3) middle AD, and (4) late AD [8].

Varenicline, a partial agonist for the α4β2 nicotinic acetylcholine receptor subtype (nACH), is used for smoking cessation. Moreover, previous randomized controlled trials (RCTs) reported that varenicline effectively improved AD outcomes [9,10,11]. However, a previous meta-analysis (MA) consisting of nine RCTs [12] illustrated that varenicline could not improve heavy drinking days but decreased alcohol consumption. In addition, the MA performed by Gandhi et al. (2020) [13] showed that varenicline did not decrease the percentage of heavy drinking days, number of drinks per drinking day, or percentage of abstinent days. 

However, results obtained from RCTs remain inconsistent, possibly due to the study design, small number of participants, study population, and intervention periods. Moreover, current studies on varenicline treatment in AD have reported controversial results. Previous studies [14,15,16] have shown that varenicline has a positive effect on the reduction of AUDs, while Hurt et al. (2018) [11], Verplaetse et al. (2016) [17], and de Bejczy et al. (2015) [18] reported no significant differences compared with placebo. 

In their studies, Oon-Arom (2019) [12] and Gandhi (2020) [13] did not investigate alcohol craving outcomes, adverse effects, or meta-regression. Therefore, this study provides an update to the previously published MA findings and meta-regression effects of varenicline in patients with AD to address the knowledge gaps in these studies. 

The objectives of this study were to conduct an updated systematic review (SR), MA, and meta-regression analyses of the effectiveness of varenicline against AD and assess the safety of varenicline compared to placebo in patients with AD.

## 2. Materials and Methods

**Figure 1:**
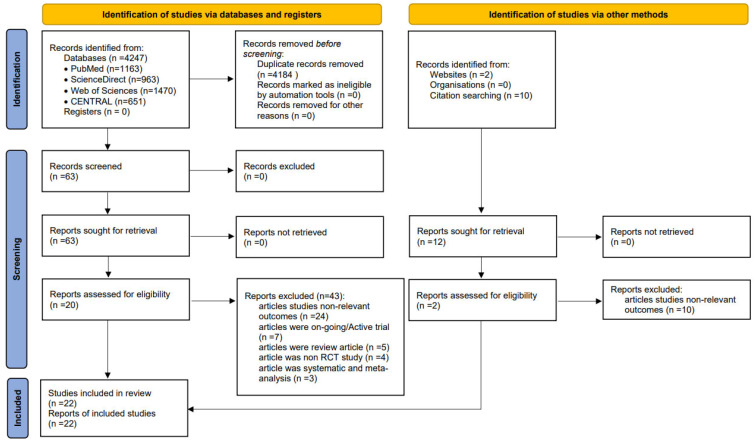
A PRISMA flow diagram describing the selection process for identifying included studies.

This study is a SR and MA conducted according to the Cochrane handbook [19]. Our study follows the preferred reporting items for systematic reviews and meta-analysis (PRISMA) checklist (Supplementary Materials) [20] and our protocol (Appendix A). The search steps are illustrated in the PRISMA flowchart (Figure 1).

### 2.1. Data Sources and Search Strategy 

RCTs comparing varenicline with placebo for AD were identified by searching the databases of PubMed, Cochrane Library, ScienceDirect, Web of Science, and ThaiLis from inception to March 2022. The following MeSH terms were searched: varenicline, alcohol, ethanol, alcohol use disorder, heavy drinkers, addiction, dependence, abuse, craving, alcoholism, and abstinence. A historical search of the reference lists of relevant systematic and narrative reviews was undertaken. Historical search refers to searching for trial reports in databases that may not retrieve all relevant available studies. Reports may either be missing from the database or have not been adequately indexed due to lack of detail in titles and abstracts. Some reports are only published as abstracts in conference proceedings. Hand searching is the task of searching through medical journals or conference publications for reports of controlled trials that are not indexed in major electronic databases. RCTs evaluating the effects of varenicline in AD treatment were eligible. Articles that were not related to outcomes of interest were excluded. There were no limitations concerning language, place, and time.

### 2.2. Study Selection and Data Extraction

Two authors (R.S. and P.P.) independently screened the titles and abstracts of various studies to identify related articles. Then, entire articles were strictly evaluated and included in the eligible studies following the predefined eligibility criteria. For a study to be included, it had to (1) be a randomized placebo-controlled trial of varenicline in AD and (2) report outcomes’ measures in terms of abstinence rate, percentage of abstinent days, percentage of drinking days, percentage of heavy drinking days, drinks per day, drinks per drinking day, alcohol intoxication, alcohol craving evaluation, and adverse effect. 

Disagreements were discussed with a third author (W.P.) until a consensus was reached. Two authors (R.S. and P.P.) systematically extracted data using the recording forms from each included study. The following information was sought from each article: (1) the publication year, (2) country of origin, (3) study design, (4) participants (the number of enrollments, dropout, and mean age), (5) dose of varenicline, (6) treatment duration, (7) outcome measurements, and (8) adverse events (AEs). Discrepancies were resolved through discussion between the two authors or consultation with the third arbitration (WP).

### 2.3. Quality Assessment

All published reports identified as potentially relevant by the literature search were assessed for inclusion in the review. The quality of RCTs was assessed using the scale developed by Jadad et al. (1996) [21], focusing on three dimensions of internal validity, namely randomization, blinding, and patient attrition, with a possible maximum score of 5 points. Studies with a score of ≤2 were considered low quality, whereas those with ≥3 were high quality.

Moreover, this study assessed the risk of bias (ROB) recommended by the Cochrane handbook for SRs of interventions [22]. The following ROB domains were considered: (1) random sequence generation, (2) allocation concealment, (3) blinding of participants and personnel, (4) blinding of outcome assessment, (5) incomplete outcome data, (6) selective reporting, and (7) other bias. The bias in each domain was judged as low risk, high risk, or unclear ROB.

### 2.4. Outcomes and Statistical Analysis 

We selected the outcomes included in ICD-11, namely substance dependence, harmful pattern of substance use, episode of harmful substance use, intoxication, and substance withdrawal. The primary outcome was an evaluation of the varenicline efficacy in AD treatment by assessing abstinence rate, percentage of drinking days, percentage of heavy drinking days, percentage of very heavy drinking days, percentage of abstinent days, drinks per day, drinks per drinking day, and alcohol craving using questionnaires, such as the Penn alcohol craving scale (PACS) and the alcohol urge questionnaire (AUQ). The secondary outcome was varenicline safety, which included AEs. The outcomes reported by the dichotomous scale were estimated using the relative risk (RR) with a 95% confidence interval (CI). Moreover, the standardized mean difference (SMD) was used to estimate the treatment effects for continuous parameters. We utilized SMD since the included studies had differences in the baseline characteristics of participants and varying durations.

Two statistical models were used for the analysis of the results: the fixed-effects model and random-effects model. The former was used when there was no significant difference among the studies included in the MA, and the latter, specifically the DerSimonian and Laird random-effects model, was used when there was a significant level of heterogeneity between the studies. The level of heterogeneity was estimated using the I^2^ value, where I^2^ < 50% indicated low heterogeneity and I^2^ = 50% or higher indicated high heterogeneity [23].

Publication bias was assessed using Eager’s weighted-regression statistics and visual inspection of funnel plots [24,25]. The DerSimonian and Laird random-effects model [26] was employed for all analyses. Statistical analysis was performed using Stata software version 14 (StataCorp, College Station, TX, USA) and Review Manager (RevMan) version 5.3.5.

Sensitivity analysis was performed by changing the effect model to ensure robustness of the results [27]. In addition, we conducted subgroup analyses based on four factors, namely duration of treatment, dose of varenicline, levels of alcohol addiction before enrollment, and AD participants alone versus those who are smokers.

Meta-regression analysis was conducted to evaluate the associations between the effect size and potential modifier variables, including dose and duration of varenicline treatment. We also performed a weighted fixed-effect meta-regression analysis using the unrestricted maximum likelihood model.

## 3. Results

### 3.1. Study Search and Selection

The literature search and selection processes are summarized in Figure 1. A systematic search through electronic databases yielded a total of 4259 articles, of which 75 potentially relevant articles were identified by title and abstract screening through a systematic literature search. These articles were selected for a full text review. Fifty-three articles were excluded based on non-relevant outcomes (n = 34), ongoing trials (n = 7), review articles (n = 5), non-RCTs (n = 4), and SR and MA (n = 3). The full texts of clinical studies were reviewed and no study was excluded in this step. Therefore, 22 articles on RCTs were eligible and included in the SR and MA [9,10,11,14,15,16,17,18,28,29,30,31,32,33,34,35,36,37,38,39,40,41].

### 3.2. Study Characteristics

The total number of participants among the included studies was 1421 (720 in the varenicline group and 701 in the placebo group). The number of participants in the included studies ranged from 10 to 200. Moreover, all included studies enrolled participants aged ≥18 years, with a mean age of 38.55 ± 6.40 years. The treatment period ranged between 8 and 112 days. Eighteen studies were performed on AUD patients, whereas four studies were on AUD with smoking patients. The dose of varenicline ranged from 1 to 2 mg/day. We included previous studies that investigated the effect of varenicline on AD in all patient groups, among which two studies were conducted on heavy alcohol drinkers with depression and 35 were on patients with schizophrenia who were both alcoholics and smokers [35]. Other characteristics of the included studies are presented in Appendix B.

### 3.3. ROB in Included Trials 

**Figure 2:**
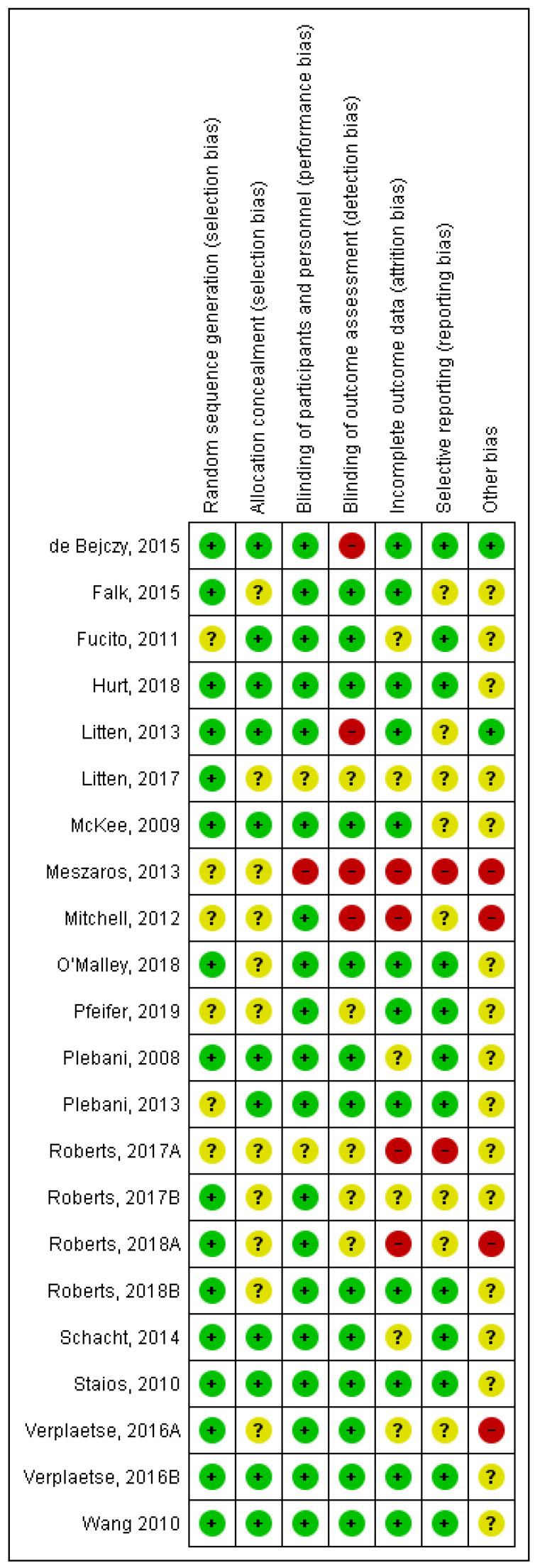
Risk of bias summary from individual studies (+, low risk; −, high risk; ?, unclear) [9,10,11,14,15,16,17,18,28,29,30,31,32,33,34,35,36,37,38,39,40,41].

The ROB assessment is shown in Figure 2. All studies that clarified the randomization were described as RCTs. Most of the described methods were of random sequence generation and allocation concealment. Hence, most trials were judged to have a low ROB in these domains. Moreover, blinding of participants and outcome assessors were found in 19 studies. One study was described as single-blinded. There was no attrition among all studies, hence, all had a low ROB in this domain. All studies described outcomes specified in the Materials and Methods Section 2.3 with low ROB.

The methodological quality of the studies was generally high, with the Jadad score ranging from 3 to 5. Only one study by Meszaros et al. (2013) [35] was given a score of two since it was not defined as a double-blind study and the randomization process was not appropriately described.

### 3.4. Clinical Outcomes 

#### 3.4.1. Primary Outcomes

##### Abstinence Rate

The pooled results of two RCTs [31,41], including 245 patients, showed no significant difference in the abstinence rate between varenicline and placebo (RR = 0.70; 95% CI: 0.21–2.35). In addition, the result of the test for heterogeneity between the studies was not significant (*p* = 0.59; I^2^ = 0.0%) (Table 1).

##### Percentage of Abstinent Days

The pooled results of four studies [10,17,18,31] (n = 396) contributing to the MA showed that varenicline significantly increased the percentage of abstinent days compared with placebo (SMD: 4.20; 95% CI: 0.21, 8.19). Heterogeneity was observed among these studies for this outcome (*p* < 0.01; I^2^ = 99.0%) (Table 1).

##### Percentage of Drinking Days

The aggregated results of two RCTs that included 66 patients with AD showed no significant difference in the percentage of drinking days between varenicline and placebo (SMD: −0.10; 95% CI: −0.58, 0.38). The test for heterogeneity was not significant (*p* = 0.90; I^2^ = 0.0%) (Table 1).

##### Percentage of Heavy Drinking Days

The pooled analyses from 11 articles [10,11,14,16,18,29,30,31,34,37,41] (n = 198) indicated no significant difference in percentage of heavy drinking days between varenicline and placebo groups (SMD: −0.07 days; 95% CI: −0.85, 0.71; *p* = 0.87) and heterogeneity was found across the trials (*p* < 0.00001; I^2^ = 96%) (Table 1).

##### Drinks per Day

Seven RCTs [10,11,28,31,32,35,36] (n = 414) reported drinks per day outcome obtained from 414 participants with pertinent AD. The pooled results of SMD revealed that the varenicline-treated group showed a significant decrease in the number of drinks per day compared with the placebo group (SMD: −0.23; 95% CI: −0.43, −0.04; *p* = 0.02). Considerable heterogeneity was not found among RCTs (*p* < 0.93; I^2^ = 0.0%) (Table 1).

##### Drinks per Drinking Day

The pooled results from three studies [11,18,31] that included 424 relevant patients with AD showed a significant reduction in the varenicline-treated group with SMD = −0.24 (95% CI: −0.44, −0.05; *p* = 0.02). There was no heterogeneity among these studies (*p* = 0.31; I^2^ = 16%) (Table 1).

##### Alcohol Intoxication

Three RCTs [9,38,40] that included 100 patients with AD reported alcohol intoxication outcomes. Although the results indicated that varenicline decreased alcohol intoxication, the difference was not statistically significant (SMD −0.87; 95% CI: −1.76, 0.03; *p* = 0.06). A random-effect was applied based on the heterogeneity found across the studies (*p* = 0.006; I^2^ = 76%) (Table 1).

##### Alcohol Craving Evaluation

Alcohol craving was evaluated using four different types of questionnaires: the obsessive-compulsive drinking scale (OCDS), PACS, AUQ, and the visual analog scale (VAS). The scores of the questionnaires ranged from 0 to 100. Six studies used the OCDS questionnaire and the pooled analysis showed no significant difference in alcohol craving between the varenicline and placebo groups (n = 341; SMD: −0.25; 95% CI: −0.72, 0.22; *p* = 0.22; I^2^ = 73%). The VAS showed that the varenicline-treated group had decreased alcohol craving but the difference was not statistically significant compared to the placebo group (n = 182; SMD: −0.26; 95% CI: −0.55, 0.04; *p* = 0.09; I^2^ = 0.0%).

In contrast, the PACS and AUQ questionnaires showed positive results for the varenicline-treated group. The pooled analysis using the PACS questionnaire indicated a significant reduction in alcohol craving in the varenicline-treated group compared with that of the placebo group (n = 285; SMD: −0.35; 95% CI: −0.59, −0.12; *p* = 0.003; I^2^ = 42%). The AUQ questionnaire also showed a significant reduction in alcohol craving in the varenicline-treated group compared with that of the placebo group (n = 337; SMD: −1.41; 95% CI: −2.12, −0.71; *p* < 0.0001), with evidence of heterogeneity (I^2^ = 87%; *p* < 0.00001) (Table 1).

#### 3.4.2. Secondary Outcome: AE

The pooled analyses showed that the varenicline-treated group was more likely to experience AEs in the gastrointestinal system, for instance, nausea or vomiting (RR 2.31; 95% CI: 1.81, 2.96) and abdominal pain (RR 3.82; 95% CI: 1.23, 11.84). In addition, central nervous system (CNS) AEs were associated with varenicline, including vivid dreams or nightmares (RR 1.89; 95% CI: 1.33, 2.69) (Table 2).

#### 3.4.3. Sensitivity Analysis

In this study, the sensitivity analysis was performed by changing the effect model to establish the sensitivity of each outcome. The results were similar to those of the main analysis; this confirmed that the main results were robust and reliable.

#### 3.4.4. Subgroup Analysis

We also conducted a subgroup analysis to reinforce the results of the main MA. This analysis was divided into four categories: (1) varenicline dose (1 and 2 mg per day), (2) duration of treatment (<30 days, 30–90 days, and >90 days), (3) alcohol consumption level (very high, high, medium, low, and no daily alcohol intake), and (4) participant characteristics (AD alone vs. AD with smoking).

The results showed that the low dose (1 mg/day) of varenicline significantly decreased alcohol intoxication but there was no significant difference in alcohol craving as evaluated by AUQ scores compared with that of the placebo group. The treatment with varenicline for 30–90 days did not improve alcohol craving as evaluated by VAS scores but there was a significant improvement in alcohol craving as evaluated by PACS scores for treatment duration >90 days. Additionally, the durations <30 days, 30–90 days, and >90 days resulted in non-significant decreases in percentage of abstinent days, drinks per day, and drinks per drinking day. The subgroup analysis of alcohol consumption levels indicated that varenicline had significant effects on reducing heavy drinking days, alcohol intoxication, and drug compliance for participants with low levels of alcohol consumption.

The subgroup analysis based on participant characteristics showed that varenicline treatment significantly improved alcohol craving scores as evaluated by PACS, AUQ, and VAS, as well as alcohol intoxication and drug compliance outcomes for participants with both AD alone and AD with smoking. The overall and stratified analysis results are presented in Appendix C.

#### 3.4.5. Meta-Regression

Meta-regression was used to evaluate the association between primary outcomes and the duration of varenicline administration. The results from the random-effect meta-regression showed three significant associations between duration of varenicline use and alcoholic outcomes, including percentage of heavy drinking days (slope = −2.64; 95% CI: −0.86, −0.07; *p* = 0.025), AUQ (slope = −2.41; 95% CI: −1.62, −0.01; *p* = 0.047), and alcohol intoxication (slope = −7.65; 95% CI: −0.82, −0.23; *p* = 0.017). 

However, varenicline duration was not associated with percentage of abstinent days (slope = −0.54; 95% CI: −5.91, 4.59; *p* = 0.644), alcohol craving (OCD) (slope = −0.64; 95% CI: −0.23, 0.15; *p* = 0.558), alcohol craving (PACS) (slope = −0.69; 95% CI: −2.08, 1.86; *p* = 0.614), and drug compliance (slope = 0.19; 95% CI: −2.73, 2.81; *p* = 0.883) (Appendix D).

#### 3.4.6. Publication Bias

Publication bias was evaluated using the funnel plot method. The results indicated that all outcomes had no publication bias, except for the percentage of abstinent days outcome. Another publication bias was assessed using the Egger’s test, which showed no publication bias in four outcomes: percentage of heavy drinking days (intercept = −0.75; SE = 3.75; 95% CI = −9.10, 7.61; *t* = −0.20; *p* = 0.846), drinks per day (intercept = −0.02; SE = 0.53; 95% CI = −1.33, 1.28; *t* = −0.05; *p* = 0.965), alcohol craving (OCD) (intercept = 0.22; SE = 2.44; 95% CI = −6.56, 7.01; *t* = 0.09; *p* = 0.932), and alcohol craving (VAS) (intercept = 0.71; SE = 0.88; 95% CI = −2.10, 3.53; *t* = 0.81; *p* = 0.480). However, it is apparent that publication bias was found in the alcohol craving (AUQ) outcome (intercept = −7.22; SE = 1.88; 95% CI = −11.68, −2.77; *t* = −3.83; *p* = 0.006).

## 4. Discussion

Varenicline has been shown to have potential benefits in reducing alcohol consumption in patients with AD. Our updated SR and MA aimed to summarize the available clinical evidence regarding the efficacy and safety of varenicline for AD treatment. The results indicated that varenicline had a positive impact on measures, such as percentage of abstinent days, drinks per day, drinks per drinking day, and alcohol craving. Although the treatment was well tolerated, the incidence of serious AEs was not reported. Meta-regression analysis suggested an association between varenicline dose and outcomes, such as percentage of heavy drinking days, AUQ score, and alcohol intoxication. However, it is important to note that our study included a larger number of trials and participants than did previous SRs [12,13]. Further well-designed RCTs with larger sample sizes and longer treatment periods are needed to confirm the results and assess the overall safety and efficacy of varenicline for AD treatment.

The mechanism of lowering alcohol cravings remains unclear but may be related to the nicotinic receptor. Davis et al. (2006) reported that alcohol acts on nicotine receptors, resulting in the alcohol craving and drinking behavior observed in both alcoholics and smokers. Varenicline had inhibitory effects on nACH, possibly reducing cravings for both cigarettes and alcohol [35,38].

Our results are consistent with those of Oon-Arom (2019) [12], who analyzed nine RCTs and showed that varenicline reduced the percentage of heavy drinking days compared to placebo but this difference was not significant. However, varenicline significantly improved the consumption of drinks per day. Results from this MA demonstrated that varenicline significantly reduced the consumption of drinks per drinking day. The safety outcomes of the previous study were not assessed, however, the acceptability test using the dropout rates method was conducted. The findings showed that the main reason for requesting to terminate the study was adverse reactions from varenicline use. In contrast to this study, there was no significant difference in the AE of treatment with varenicline in any dosage range or duration compared to placebo.

Meanwhile, the comparison with the MA of Gandhi et al., (2020) [13] that included 10 studies showed that the outcome of percentage of heavy drinking days was significantly reduced with varenicline but did not significantly differ from placebo. The results of drinks per drinking day and the percentage of abstinent days were inconsistent with those of this MA. Varenicline reduced the amount of alcohol consumption on drinking days or the abstinent days, however, there were no differences from the placebo group.

Erwin et al., (2014) [42] reported that varenicline affected alcohol outcomes, including decreasing the amount of alcohol consumption and duration of drinking. There are two possible mechanisms to explain the effects of varenicline: (1) varenicline affected the rewarding system, thereby lowering alcohol craving, and (2) varenicline exacerbated the negative effects of alcohol intake, such as headache and dizziness. 

Although there were no reports of severe AEs in the varenicline and placebo groups, our MA found that varenicline treatment is accompanied with gastrointestinal effects (nausea/vomiting and abdominal pain) and other AEs associated with CNS (vivid dreams or nightmares).

The possible AE mechanism may be due to varenicline being a partial agonist that activates nACH. Varenicline is a partial agonist of nACH on neurons and stimulates dopamine release, which may affect the chemoreceptor trigger zone, resulting in flatulence, visceral pain, nausea, and vomiting [43,44]. 

These AEs were found to be comparable to those reported with other medications used for alcoholism treatment, such as naltrexone, acamprosate, and disulfiram. Naltrexone is an opioid receptor antagonist that has been shown to reduce alcohol craving and improve the success of alcohol abstinence. Common side effects of naltrexone include nausea, headache, dizziness, and liver problems. However, it is generally considered safe and well tolerated [45]. Acamprosate is another medication that has been used to treat alcoholism. The most common side effects include diarrhea, headache, and nausea [46]. Disulfiram treatment is associated with unpleasant symptoms, such as flushing, sweating, and headache, after drinking alcohol.

Another study comparing varenicline to naltrexone for the treatment of alcoholism found similar AEs, including nausea, headache, and insomnia. However, the study also found that varenicline was associated with a higher incidence of constipation than naltrexone [47,48]. However, this MA found that the incidence of constipation in the varenicline-treated group was not different from that in the placebo group. It is important to note that the AEs of varenicline may vary depending on the dose and duration of treatment. Additionally, the reported AEs should be considered in light of the potential benefits of varenicline for reducing alcohol craving and consumption.

In addition, these actions may decrease non-rapid eye movement (NREM) sleep. In other words, varenicline may competitively inhibit the binding of acetylcholine receptors. Generally, acetylcholine prevents REM sleep. Therefore, varenicline is associated with the features of REM sleep, with wakefulness as nightmares [44,49]. 

However, there have been concerns about the safety of varenicline, particularly with regard to the presence of nitrosamines, which are carcinogenic compounds. In recent years, the presence of high levels of nitrosamines in varenicline has led to regulatory actions in several countries, including delays in approvals or restrictions on its use. In 2020, the European Medicines Agency issued a warning about the potential presence of nitrosamines in varenicline and recommended that patients who are taking the medication should continue to do so, as the benefits of quitting smoking outweigh the potential risks from nitrosamines [50]. Moreover, Lang (2023) [51] reported that prescriptions of varenicline were reduced because of this limitation and that the drug was subsequently removed from the market in 2021. The substantial decrease in varenicline use after the drug’s recall represents potential lost opportunities for nicotine cessation with likely immediate and long-term adverse health outcomes. This may further affect varenicline use for AD.

The results of the meta-regression analysis herein suggest that the duration of varenicline administration is associated with several alcoholic outcomes. Specifically, the results showed significant associations between varenicline use and a decrease in percentage of heavy drinking days, a decrease in AUQ, and a decrease in alcohol intoxication. Previous studies have also explored the effects of varenicline on alcohol consumption. An RCT by Mitchell et al. (2012) [28] found that varenicline was effective in reducing alcohol consumption and craving in heavy-drinking smokers. Another study by McKee et al. (2009) [9] showed that varenicline reduced alcohol craving and the number of drinks consumed per drinking day in heavy-drinking individuals with a history of AD.

However, the results of the current study suggest that varenicline may not have a significant effect on other outcomes, such as percentage of abstinent days, alcohol craving as measured by OCD, alcohol craving as measured by PACS, and drug compliance. This is consistent with previous findings by Gandhi et al., (2020) [13] who found that varenicline had no significant effect on the number of abstinent days from alcohol in heavy-drinking individuals.

The current evidence suggests that varenicline may have potential in reducing certain aspects of alcohol consumption, as demonstrated by the findings in the present study. However, despite these promising findings, there is still a need for more research to fully understand the effects of varenicline on different alcoholic outcomes and to determine the optimal duration of varenicline administration. Moreover, further research is needed to fully understand the effect size and any potential side effects associated with its use. Additionally, there may be other factors, such as comorbidities or individual patient characteristics, that can impact the effectiveness of varenicline as a treatment option.

The strengths of this SR and MA study are as follows: (1) this study is an updated MA that included 22 RCTs, most of which were of high quality and had low ROB; (2) this MA performed a systematic search through five international databases and a Thai database, along with a manual search for unpublished trials; (3) subgroup analysis and meta-regression were performed to determine the effects of variable on outcomes; (4) AEs were pooled by MA, whereas previous SR and MA studies [12,13] did not perform subgroup analysis and meta-regression. 

Nevertheless, this study has some limitations: (1) most of the included trials were conducted with a small number of participants and a short-term duration; (2) the studies included various population characteristics and intervention periods; (3) most of the selected RCTs did not report underlying disease and contaminant medication; (4) some outcomes showed publication bias when evaluated using Egger’s test and funnel plot. These may be the root cause of the inconsistent results along with the heterogeneity; (5) the abstinence rate and percentage of drinking days were pooled from two RCTs; as only two RCTs may not have enough power to detect significant differences or to make strong conclusions, the results should be interpreted with caution and further research is needed to replicate and validate the findings.

## 5. Conclusions

The results from this MA indicated that varenicline is effective for the percentage of abstinent days, drinks per day, drinks per drinking day, alcohol intoxication, and alcohol craving outcomes. Notably, there were no reports of severe effects in both varenicline and placebo-treated groups. The results of this MA suggest that varenicline may have potential as a treatment for AD. However, large-scale, long-term RCTs on the effects of varenicline on AD in combination with other treatments or network meta-analysis to compare treatment efficacy in AD remain warranted.

## Figures and Tables

**Table 1:** Outcome comparison between varenicline and placebo.

Outcomes	Main Analysis	References
Abstinent rate	RR = 0.70 (95% CI: 0.21, 2.35; *p* = 0.56); I^2^ = 0.0% (FE)	[31,41]
Percentage of abstinent days	SMD = 4.20 days (0.21, 8.19; *p* = 0.04); I^2^ = 99.0% * (RE)	[10,17,18,31]
Percentage of drinking days	SMD = −0.10 days (−0.58, 0.38; *p* = 0.69); I^2^ = 0.0% (FE)	[11]
Percentage of heavy drinking days	SMD = −0.07 days (−0.85, 0.71; *p* = 0.87); I^2^ = 96.0% (RE)	[10,11,14,16,18,29,30,31,34,37,41]
Drinks per day	SMD = −0.23 drinks (−0.43, −0.04; *p* = 0.02); I^2^ = 0.0% * (FE)	[10,11,28,31,32,35,36]
Drinks per drinking day	SMD = −0.24 drinks (−0.44, −0.05; *p* = 0.01); I^2^ = 16.0% * (FE)	[11,18,31]
Alcohol intoxication	SMD = −0.87 drinks (−1.76, 0.03; *p* = 0.06); I^2^ = 76.0% (RE)	[9,39,40]
Alcohol craving (OCDS scale)	SMD = −0.25 (−0.72, 0.22; *p* = 0.22); I^2^ = 73.0% (RE)	[16,18,34,36]
Alcohol craving (PACS scale)	SMD = −0.35; 95% CI −0.59, −0.12; *p* = 0.003; I^2^ = 42.0% * (FE)	[31,37,41]
Alcohol craving (AUQ scale)	SMD = −1.41 (−2.12, −0.71; *p* < 0.00001); I^2^ = 87.0% * (RE)	[9,15,17,33,39,40]
Alcohol craving (VAS)	SMD = −0.26 (−0.55, 0.04; *p* = 0.09); I^2^ = 0.0% (FE)	[32,33,35,36,41]

Abbreviations: RR: Risk ratio; SMD: Standard mean difference; OCDS: obsessive-compulsive drinking scale; PACS: Penn alcohol craving scale; AUQ: alcohol urge questionnaire; VAS: visual analog scale; CI: Confidence interval; FE: Fixed-effect model; RE: Random-effect model; * *p* ≤ 0.05.

**Table 2:** Results of studies reporting adverse effects.

Adverse Effect (No. of Studies)	No of Events/ No. of Patients in Varenicline Groups (%)	No of Events/ No. of Patients in Placebo Groups (%)	Pooled Risk Ratio (95% CI)	I^2^	P^a^
(1) Gastrointestinal system					
Nausea/Vomiting (14)	152/432 (35.19)	66/425 (15.5)	2.31 (1.81, 2.96) *	0.0%	0.63
Constipation (5)	14/168 (8.33)	7/152 (4.61)	1.68 (0.75, 3.73)	32%	0.21
Flatulence (4)	7/72 (9.72)	9/51 (17.65)	0.66 (0.3, 1.45)	0.0%	0.94
Abdominal pain (3)	12/91 (13.19)	3/98 (3.06)	3.82 (1.23, 11.84) *	0.0%	0.88
Diarrhea (5)	28/265 (10.57)	27/282 (9.57)	1.11 (0.68, 1.82)	49%	0.1
Dry mouth (4)	19/190 (10.00)	14/180 (7.78)	1.18 (0.64, 2.16)	73%	0.01
Abdominal discomfort (1)	4/77 (5.19)	4/83 (4.82)	1.08 (0.28, 4.16)	N/A	N/A
Dysgeusia (1)	6/96 (6.25)	1/101 (0.99)	6.31 (0.77, 51.47)	N/A	N/A
Heartburn (1)	3/12 (25.00)	0/12 (0.00)	7.0 (0.40, 122.44)	N/A	N/A
(2) Nervous system					
Headache (9)	63/344 (18.31)	61/359 (16.99)	1.09 (0.80, 1.49)	0.0%	0.51
Vivid dream/nightmares (11)	72/392 (18.37)	38/389 (9.77)	1.89 (1.33, 2.69) *	0.0%	0.56
Erratic behavior (2)	0/49 (0.00)	0/30 (0.00)	N/A	N/A	N/A
Insomnia (8)	35/341 (10.26)	27/337 (8.01)	1.25 (0.78, 2.01)	0.0%	0.98
Suicidal thoughts (4)	1/87 (1.15)	2/66 (3.03)	0.58 (0.09, 3.90)	0.0%	0.57
Depression (6)	23/222 (10.36)	20/237 (8.43)	1.22 (0.7, 2.13)	0.0%	0.89
Appetite change (2)	7/73 (9.59)	7/77 (9.09)	1.06 (0.4, 2.79)	31%	0.23
Sleep problem (4)	11/136 (8.09)	10/141 (7.09)	1.14 (0.52, 2.49)	16%	0.31
Anxiety (5)	16/207 (7.73)	18/218 (8.26)	0.94 (0.5, 1.75)	4.0%	0.39
Drowsiness (2)	11/76 (14.47)	8/79 (10.13)	1.42 (0.6, 3.36)	56%	0.13
Vertigo (1)	7/77 (9.09)	6/83 (7.23)	1.26 (0.44, 3.58)	N/A	N/A
Pyrexia (1)	1/77 (1.30)	6/83 (7.23)	0.18 (0.02, 1.46)	N/A	N/A
Seizure (1)	1/23 (4.35)	0/24 (0.00)	3.13 (0.13, 73.01)	N/A	N/A
Agitation (3)	13/152 (8.55)	18/156 (11.54)	0.77 (0.4, 1.47)	0.0%	0.5
Anger (1)	2/33 (6.06)	0/31 (0.00)	4.71 (0.23, 94.31)	N/A	N/A
Aggression (1)	1/33 (3.03)	0/31 (0.00)	2.82 (0.12, 66.82)	N/A	N/A
Somnolence (1)	6/96 (6.25)	13/101 (12.87)	0.49 (0.19, 1.23)	N/A	N/A
Dizziness (1)	11/96 (11.46)	6/101 (5.94)	1.93 (0.74, 5.01)	N/A	N/A
Irritability (2)	13/101 (12.87)	8/106 (7.55)	1.69 (0.76, 3.76)	58%	0.12
Hostility (1)	6/96 (6.25)	4/101 (3.96)	1.58 (0.46, 5.42)	N/A	N/A
Auditory visual hallucination (1)	0/5 (0.00)	1/5 (20.00)	0.33 (0.02, 6.65)	N/A	N/A
Paranoia (1)	0/5 (0.00)	1/5 (20.00)	0.33 (0.02, 6.65)	N/A	N/A
(3) Eye/ear/nose/throat (EENT) and respiratory system					
Difficulty breathing (2)	2/49 (4.08)	1/30 (3.33)	1.29 (0.17, 9.67)	0.0%	0.88
Blurred vision (2)	3/87 (3.45)	2/91 (2.20)	1.57 (0.27, 9.05)	0.0%	0.71
Rhinorrhea (1)	8/64 (12.50)	4/67 (5.97)	2.09 (0.66, 6.61)	N/A	N/A
Shortness of breath (1)	1/40 (2.50)	1/20 (5.00)	0.5 (0.03, 7.59)	N/A	N/A
Nasopharyngitis/Upper respiratory tract infection (3)	27/196 (13.78)	38/208 (18.27)	0.76 (0.48, 1.19)	25%	0.26
Cough (2)	1/96 (1.04)	6/104 (5.77)	0.25 (0.04, 1.45)	36%	0.21
(4) Musculoskeletal system and skin					
Fatigue (2)	31/173 (17.92)	20/184 (10.87)	1.65 (0.98, 2.78)	0.0%	0.43
Arthralgia (2)	13/173 (7.51)	16/184 (8.70)	0.86 (0.43, 1.75)	47%	0.17
Back pain (2)	10/173 (5.78)	17/184 (9.24)	0.63 (0.29, 1.33)	0.0%	0.74
Body ache (1)	5/19 (26.32)	4/21 (19.05)	1.38 (0.43, 4.4)	N/A	N/A
Rash (1)	3/96 (3.13)	6/101 (5.94)	0.53 (0.14, 2.04)	N/A	N/A
(5) Circulatory system					
Chest pain (3)	1/145 (0.69)	7/131 (5.34)	0.21 (0.01, 1.19)	49%	0.16
Fast heartbeat (2)	4/49 (8.16)	1/30 (3.33)	1.97 (0.34, 11.47)	0.0%	0.68
High blood pressure (2)	2/24 (8.33)	4/26 (15.38)	0.53 (0.11, 2.47)	0.0%	0.95
Raynaud phenomenon (1)	0/5 (0.00)	1/5 (20.00)	0.33 (0.02, 6.65)	N/A	N/A
(6) Urinary and reproductive system					
Bright urine (1)	3/23 (13.04)	1/24 (4.17)	3.13 (0.35, 27.96)	N/A	N/A
Gynecological bleeding (1)	1/23 (4.35)	2/24 (8.33)	0.52 (0.05, 5.37)	N/A	N/A

Abbreviations: CI: Confidence interval; P^a^: *p*-value for heterogeneity; N/A: not available. * *p* ≤ 0.05.

**Figure A1:**
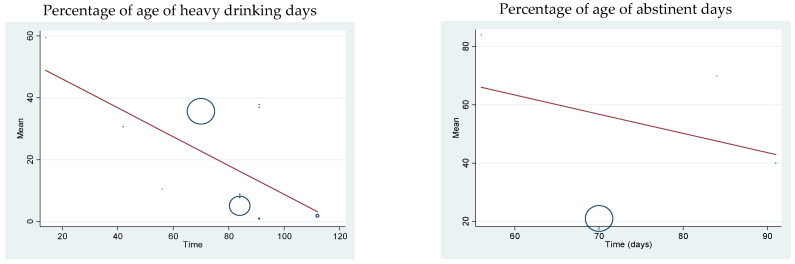

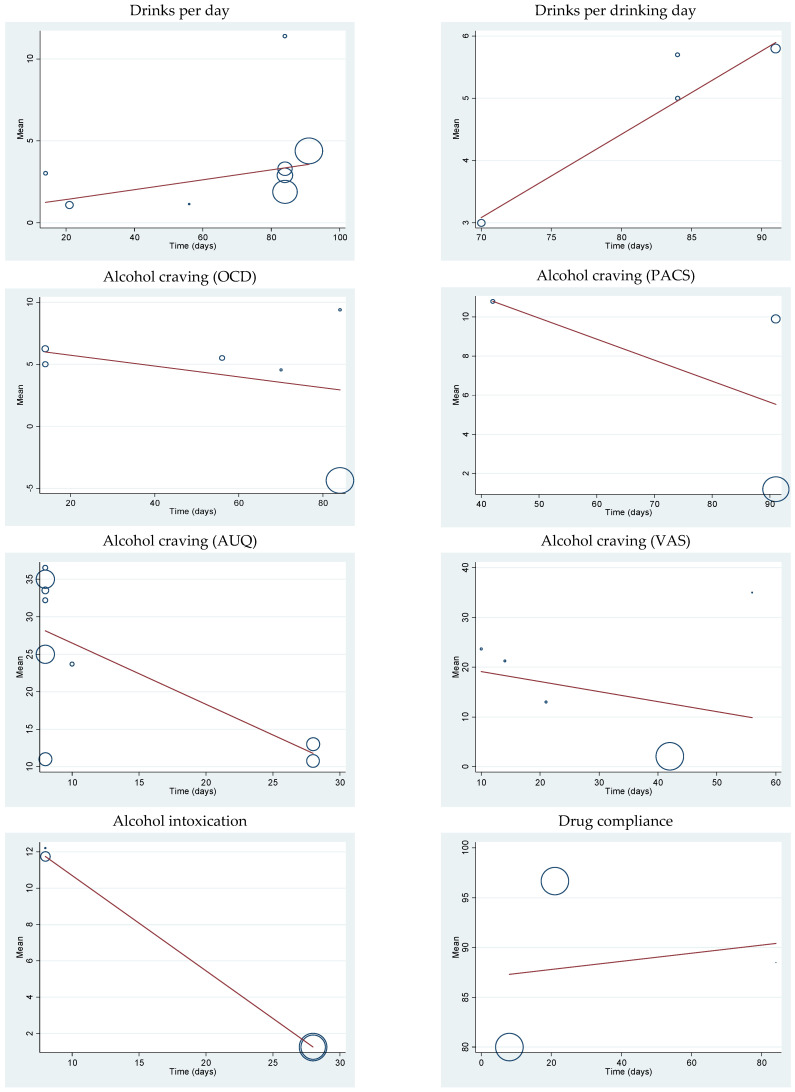
Meta-regression of varenicline efficacy on primary outcomes.

## Supplementary Materials

The following supporting information can be downloaded at: https://www.mdpi.com/article/10.3390/ijerph20054091/s1. File S1. PRISMA 2020 Checklist [20].

## Appendix A

Study Protocol

Efficacy of varenicline in the treatment of alcohol dependence: An updated meta-analysis and meta-regression.

Review Question

To perform an updated meta-analysis and meta-regression of the effects of varenicline in patients with alcohol dependence (AD). Moreover, this study assessed the safety of varenicline use in patients with AD.

Searches

The PRISMA guidelines for conducting systematic review were followed. The databases searched were PubMed, Cochrane Library, ScienceDirect, Web of Science, and ThaiLis. The included studies were randomized controlled trials evaluating the effect and/or safety of varenicline in patients with AD.

The search terms were as follows: varenicline, alcohol, ethanol, alcohol use disorder, heavy drinkers, addiction, dependence, abuse, craving, alcoholism, and abstinence.

In addition, a historical search and hand search of references of the included articles were carried out, along with any further material identified for inclusion.

There were no limitations concerning language, place, and time.

Types of studies included

Only randomized controlled trials (RCTs) were included. The studies reported outcomes in terms of abstinence rate, percentage of abstinent days, percentage of drinking days, percentage of heavy drinking days, drinks per day, drinks per drinking day, alcohol intoxication, alcohol craving, and adverse effect.

Condition or domain studied

The meta-analysis and meta-regression investigated the use of varenicline in patients with AD.

Participants/population

Patients with AD.

Intervention(s), exposure(s)

Varenicline oral with any dose.

Comparators/control

Placebo.

Context

Primary outcome(s): abstinence rate, percentage of drinking days, percentage of heavy drinking days, percentage of very heavy drinking days, percentage of abstinent days, drinks per day, drinks per drinking day, and alcohol craving using questionnaires, such as the Penn alcohol craving scale (PACS) and the alcohol urge questionnaire (AUQ).

Secondary outcome(s): adverse events.

Data extraction (Selection and coding)

From the retrieved abstracts, the principal investigator first considered all titles of articles and selected RCTs evaluating the efficacy of varenicline in AD. The abstracts of all selected articles were assessed using an abstract evaluation form by two authors working independently. Following this, the two authors independently evaluated the full details of all selected articles using a data extraction form. The extracted data included the following items: authorship, year of publication, location/region of study, population, type of economic analysis, strategies assessed (intervention versus comparator), abstinence rate, percentage of drinking days, percentage of heavy drinking days, percentage of very heavy drinking days, percentage of abstinent days, drinks per day, drinks per drinking day, and alcohol craving using questionnaires, such as the Penn alcohol craving scale (PACS) and the alcohol urge questionnaire (AUQ), and adverse events. A third author’s opinion was sought if disagreements occurred between the two researchers.

Risk of bias (quality) assessment

The quality of the included studies assessed using the Jadad’s scale was used as a guideline to evaluate methodological quality of included studies. Risk of bias in individual studies was assessed using the risk of bias tool of Cochrane Handbook for Systematic Reviews of Interventions. The approval of the two authors for the selected extracted data was sought. The opinion of a third author was requested if a disagreement occurred between the two researchers.

Strategy for data synthesis

Data were extracted by two authors working independently and added into a table for analysis. Efficacy between the varenicline usage and the placebo groups was statistically tested using relative risk (RR) with a 95% confidence interval (CI) for the outcomes reported by the dichotomous scale. Moreover, the standardized mean difference (SMD) was used to estimate the treatment effects for continuous parameters. The heterogeneity of included studies was examined using the Q-statistic, while results were analyzed using the I-squared statistic. Random-effects modelling was used if the included studies were heterogeneous and fixed-effects modelling was used if homogeneity was found. The publication bias was examined via Eager’s weighted regression statistics and the means of funnel plot asymmetry.

Analysis of subgroups or subsets

Subgroup analysis was performed based on four factors, namely duration of treatment, dose of varenicline, alcohol consumption levels before enrollment, and AD participants alone versus those who are smokers.

Meta-regression

Meta-regression was employed to evaluate associations between the effect size and potential modifier variables, which included dose and duration of varenicline treatment. We performed a weighted fixed-effect meta-regression using the unrestricted maximum likelihood model.

Anticipated or actual start date

15 January 2020.

Anticipated completion date

31 March 2022.

Funding sources/sponsors

This research project was financially supported by Mahasarakham University (Grant Number 2565).

Conflicts of interest

None known.

Language

English.

Country

Thailand.

## Appendix B

**Table A1:** Characteristics of studies included in the meta-analysis.

Authors (Year)	Location	Study Duration	Participants	Age (Years)	Intervention/ Comparator (N)	Outcomes	AE Reported	Jadad Score
Pfeifer et al., (2019) [10]	Germany	84 days	alcohol and nicotine dependence patients (N = 28)	45.0 ± 8.12	– varenicline 2 mg/day (N = 15), – placebo (N = 13)	Percentage ofage of days without alcohol consumption, number of standardized drinks per day, percentage ofage of heavy drinking days, and alcohol craving (OCD scale)	✓	4
Roberts et al., (2017) [15]	USA	8 days	heavy drinking tobacco users (N = 30)	33.59 ± 9.86	– varenicline 2 mg/day (N = 9), – varenicline 2 mg/day with low-dose naltrexone (25 mg/day) (N = 11), – placebo (N = 10)	Alcohol craving (AUQ scale) and subjective alcohol intoxication effects	✓	5
Hurt et al., (2018) [11]	USA	84 days	drinking smokers (N = 33)	39.5 ± 11.15	– Varenicline 2 mg/day (N = 16), – Placebo (N = 17)	Number of heavy drinking days, average drinks per day, average drinks per drinking day, and number of drinking days	✓	4
Roberts et al., (2018) [38]	USA	10 days	heavy drinkers (with smokers or nonsmokers) (N = 77)	34.26 ± 9.78	– Varenicline 2 mg/day (N = 39), – Placebo (N = 38)	Alcohol-cue-induced craving (AUQ scale)	✕	3
Verplaetse et al., (2016) [17]	USA	28 days	alcohol abuse or alcohol dependence patients (N = 44)	33.78 ± 9.02	– Varenicline 1 mg/day (N = 12), – Varenicline 2 mg/day (N = 15), – Placebo (N = 17)	Alcohol craving (AUQ scale) and alcohol intoxication	✕	3
Roberts et al., (2018) [39]	USA	8 days	Alcohol abuse or dependence patients (N = 55)	34.26 ± 9.78	– Varenicline 1 mg/day (N = 20), – Varenicline 2 mg/day (N = 20), – Placebo (N = 15)	ad-libitum alcohol consumption and total number of drinks	✕	5
O’Malley et al., (2018) [14]	USA	112 days	Alcohol-dependent smokers (N = 131)	42.75 ± 11.75	– Varenicline 2 mg/day (N = 64), – Placebo (N = 67)	Percentage ofage of heavy drinking days	✓	5
Verplaetse et al., (2016) [40]	USA	8 days	Alcohol consumers (N = 60)	33.90 ± 9.88	– Varenicline 1 mg/day (N = 20), – Varenicline 2 mg/day (N = 20), – Placebo (N = 20)	Alcohol craving (AUQ scale) and number of Ad-libitum drinks in drinking period	✓	5
de Bejczy et al., (2015) [18]	Sweden	70 days	Alcohol-dependent subjects (N = 171)	55.10 ± 7.99	– Varenicline 2 mg/day (N = 86), – placebo (N = 85)	Proportion of heavy drinking days, proportion of abstaining days, alcohol consumption drinks per drinking day, and alcohol craving (OCD scale)	✓	5
Litten et al., (2017) [41]	USA	42 days	Alcohol drinkers (N = 47)	≥21 years	– Varenicline 2 mg/day (N = 23), – placebo (N = 24)	Percentage of heavy drinking days, Percentage ofage of age of subjects abstinent during the last month of treatment, and Alcohol craving (Penn alcohol craving scale (PACS))	✓	4
Mitchell et al., (2012) [28]	USA	84 days	Social smoking drinkers (N = 64)	27.0 ± 7.63	– Varenicline 2 mg/day (N = 33), – placebo (N = 31)	alcoholic drinks per week, alcohol craving (OCD scale), and cumulative alcoholic drinks consumed	✓	5
Plebani et al., (2013) [29]	USA	84 days	Alcoholism patients (N = 40)	18–70 years	– Varenicline 2 mg/day (N = 19), – Placebo (N = 21)	Rate of heavy drinking days per week and addiction severity index (ASI)	✓	4
Schacht et al., (2014) [16]	USA	14 days	Drinkers seeking treatment (N = 35)	30.25 ± 8.15	– Varenicline 2 mg/day (N = 18), – Placebo (N = 17)	percentage ofage of heavy drinking days and alcohol craving (OCD scale)	✓	4
Falk et al., (2015) [30]	USA	91 days	Alcohol dependence patients (N = 200)	48.1 ± 6.7	– Varenicline 2 mg/day (N = 99), – Placebo (N = 101)	Percentage ofage of heavy drinking days	✕	5
Litten et al., (2013) [31]	USA	91 days	Alcohol dependent patients (N = 198)	45.5 ± 12.1	– Varenicline 2 mg/day (N = 97), – Placebo (N = 101)	Percentage ofage of heavy drinking days, drinks per day, drinks per drinking day, percentage ofage of very heavy drinking days, percentage ofage of abstinent days, percentage ofage of subjects abstinent, percentage ofage of subjects with no heavy drinking days, alcohol craving (PACS scale), alcohol-related consequences (ImBIBe score), and quality of life (SF-12 physical/mental aggregate score)	✓	5
Staios (2010) [32]	USA	21 days	Treatment seeking smokers, mild drinkers (N = 24)	33.17 ± 9.91	– Varenicline 2 mg/day (N = 12), – Placebo (N = 12)	cue-induced craving after exposure to alcohol cues, self-reported changes in consumption of alcohol (drinks per day), and overall craving alcohol	✓	5
Roberts et al., (2017) [33]	USA	8 days	Depressed-alcohol heavy drinkers (N = 60)	33.75 ± 10.69	– Varenicline 1 mg/day (N = 20), – Varenicline 2 mg/day (N = 20) – Placebo (N = 20)	Ad-libitum alcohol consumption, alcohol craving before drinking (tonic craving), and alcohol craving after drinking (AUQ scale)	✕	3
Plebani et al., (2008) [37]	USA	91 days	Alcohol dependent patients (N = 40)	46.45 ± 11.4	– Varenicline 2 mg/day (N = 19), – Placebo (N = 21)	Week days of alcohol use, presence/absence of alcohol use, numbers of heavy drinking days presence/absence of heavy drinking, alcohol craving (PACS scale), and addiction severity index (ASI)	✕	4
Fucito et al., (2011) [34]	USA	56 days	Heavy drinking smokers (N = 30)	43.12 ± 8.26	– Varenicline 2 mg/day (N = 15), – Placebo (N = 15)	Percentage ofage of heavy drinking days, percentage ofage of abstinent days, alcohol craving (OCD scale), and alcohol sedating effect	✓	5
McKee et al., (2009) [9]	USA	8 days	Non-alcohol-dependent heavy drinkers (N = 20)	34.75 ± 12.4	– Varenicline 2 mg/day (N = 10), – Placebo (N = 10)	Number of drinks consumed during Ad-libitum period, alcohol craving (AUQ scale), and alcohol intoxication effects	✓	5
Meszaros et al., (2013) [35]	USA	56 days	Schizophrenia alcoholic and smoking patients (N = 10)	43.0 ± 7.0	– Varenicline 2 mg/day (N = 5), – Placebo (N = 5)	Number of standard drinks consumed per week, percentage ofage of abstinent days from alcohol a month, and alcohol craving	✓	2
Wang (2010) [36]	Canada	14 days	Tobacco dependence and Heavy alcohol users (N = 24)	36.10 ± 11.30	– Varenicline 2 mg/day (N = 13), – Placebo (N = 11)	visual analogue scale in tobacco-alcohol cues (VAS), obsessive compulsive drinking scale (OCDS), and alcoholic drinks consumed per day	✓	5

Remark: ✓ = yes, ✕ = no.

## Appendix C

**Table A2:** Results of subgroup analysis of RCTs evaluating clinical outcomes of varenicline.

Outcomes		No. of Trial	Effect Size	95% CI	I^2^ (%)	P for Effect Size	P^a^
(1) Abstinent rate							
Treatment duration (days)							
	30–90	1	0.52	0.11, 2.58	N/A	0.42	N/A
	>90	1	1.04	0.15, 7.25	N/A	0.97	N/A
Alcohol consumption level risk							
	Medium (60–89 g/d)	2	0.70	0.21, 2.35	0.0%	0.56	0.59
Participants’ characteristic							
	AD	2	0.70	0.21, 2.35	0.0%	0.56	0.59
(2) Percentage ofage of drinking days							
Treatment duration (days)							
	30–90	2	−0.10	−0.58, 0.38	0.0%	0.69	0.90
Alcohol consumption level risk							
	Medium (60–89 g/d)	2	−0.10	−0.58, 0.38	0.0%	0.69	0.90
Participants’ characteristic							
	AD	2	−0.10	−0.58, 0.38	0.0%	0.69	0.90
(3) Percentage ofage of heavy drinking days							
Treatment duration (days)							
	<30	1	0.10	−0.56, 0.77	N/A	0.76	N/A
	30–90	6	−0.29	−2.19, 1.61	98%	0.78	<0.00001
	>90	4	−0.02	−0.28, 0.23	54%	0.86	0.09
Alcohol consumption level risk							
	Medium (60–89 g/d)	8	−0.01	−0.98, 0.96	97%	0.99	<0.00001
	Low (30–59 g/d)	1	−0.79	−1.53, −0.04	N/A	0.04 *	N/A
	N/A	2	−0.08	−0.56, 0.39	0.0%	0.73	0.84
Participants’ characteristic							
	AD	7	−0.06	−1.35, 1.24	98%	0.93	<0.00001
	AD with smoking	4	−0.10	−0.37, 0.17	9.0%	0.47	0.36
(4) Percentage ofage of abstinent days							
Treatment duration (days)							
	30–90	3	5.71	−2.64, 14.07	99%	0.18	<0.00001
	>90	1	0.14	−0.14, 0.42	N/A	0.33	N/A
Alcohol consumption level risk							
	Medium (60–89 g/d)	2	8.76	−8.19, 25.70	100%	0.31	<0.00001
	Low (30–59 g/d)	1	0.05	−1.19, 1.29	N/A	0.94	N/A
	N/A	1	−0.18	−0.93, 0.56	N/A	0.63	N/A
Participants’ characteristic							
	AD	2	8.76	−8.19, 25.70	100%	0.31	<0.00001
	AD with smoking	2	−0.12	−0.76, 0.52	0.0%	0.71	0.75
(5) Drinks per day							
Treatment duration (days)							
	≤30	2	−0.03	−0.60, 0.54	0.0%	0.92	0.97
	30–90	4	−0.31	−0.61, −0.00	0.0%	0.05	0.78
	>90	1	−0.22	−0.50, 0.06	N/A	0.12	N/A
Alcohol consumption level risk							
	Medium (60–89 g/d)	2	−0.23	−0.47, 0.02	0.0%	0.07	0.99
	Low (30–59 g/d)	4	−0.13	−0.49, 0.22	0.0%	0.47	0.96
	N/A	1	−0.77	−1.55, 0.00	N/A	0.05	N/A
Participants’ characteristic							
	AD	1	−0.22	−0.50, 0.06	N/A	0.12	N/A
	AD with smoking	6	−0.24	−0.51, 0.03	0.0%	0.08	0.87
(6) Drinks per drinking day							
Treatment duration (days)							
	30–90	2	−0.25	−0.51, 0.01	44%	0.06	0.17
	>90	1	−0.24	−0.52, 0.04	N/A	0.09	N/A
Alcohol consumption level risk							
	Medium (60–89 g/d)	3	−0.24	−0.44, −0.05	16%	0.01 *	0.31
Participants’ characteristic							
	AD	2	0.17	−0.38, 0.03	0.0%	0.10	0.49
	AD with smoking	1	−0.62	−1.33, 0.08	N/A	0.08	N/A
(7) Alcohol craving (OCDS)							
Treatment duration (days)							
	<30	2	0.02	−1.53, 1.49	87%	0.98	0.005
	30–90	4	−0.33	−0.84, 0.19	72%	0.21	0.001
Alcohol consumption level risk							
	Medium (60–89 g/d)	2	−0.37	−1.00, 0.27	66%	0.26	0.09
	Low (30–59 g/d)	3	−0.02	−1.12, 1.08	87%	0.97	0.0004
	N/A	1	−0.42	−1.17, 0.33	N/A	0.27	N/A
Participants’ characteristic							
	AD	2	−0.37	−1.00, 0.27	66%	0.26	0.09
	AD with smoking	4	−0.13	−0.93, 0.66	81%	0.74	0.001
(8) Alcohol craving (PACS)							
Treatment duration (days)							
	30–90	1	−0.02	−0.59, 0.55	N/A	0.95	N/A
	>90	2	−0.42	−0.68, −0.16	46%	0.001 *	0.17
Alcohol consumption level risk							
	Medium (60–89 g/d)	2	−0.28	−0.53, −0.03	0.0%	0.03 *	0.32
	N/A	1	−0.84	−1.49, −0.19	N/A	0.01 *	N/A
Participants’ characteristic							
	AD	3	−0.35	−0.59, −0.12	42%	0.003 *	0.18
(9) Alcohol craving (AUQ)							
Varenicline dose							
	1 mg/day	3	−1.11	−2.35, 0.13	88%	0.08	0.0002
	2 mg/day	3	−1.59	−2.56, −0.62	89%	0.001 *	<0.00001
Treatment duration (days)							
	<30	6	−1.41	−2.12, −0.71	87%	<0.0001 *	<0.00001
Alcohol consumption level risk							
	Medium (60–89 g/d)	4	−1.41	−2.26, −0.56	88%	0.001 *	<0.00001
	Low (30–59 g/d)	2	−1.58	−3.99, 0.82	91%	0.20	0.0006
Participants’ characteristic							
	AD	4	−1.72	−2.65, −0.79	89%	0.0003 *	<0.00001
	AD with smoking	2	−0.46	−0.87, −0.06	0.0%	0.02 *	0.78
(10) Alcohol craving (VAS)							
Treatment duration (days)							
	<30	3	−0.34	−0.70, 0.01	0.0%	0.06	0.72
	30–90	2	−0.07	−0.59, 0.45	0.0%	0.79	0.88
Alcohol consumption level risk							
	Medium (60–89 g/d)	1	−0.05	−0.62, 0.52	N/A	0.86	N/A
	Low (30–59 g/d)	4	−0.33	−0.67, 0.01	0.0%	0.06	0.86
(11) Alcohol intoxication							
Varenicline dose							
	1 mg/day	1	−0.88	−1.65, −0.10	N/A	0.03 *	N/A
	2 mg/day	3	−0.87	−1.76, 0.03	76%	0.06	0.006
Treatment duration (days)							
	<30	3	−0.87	−1.76, 0.03	76%	0.06	0.006
Alcohol consumption level risk							
	Medium (60–89 g/d)	2	−0.50	−1.25, 0.25	62%	0.19	0.07
	Low (30–59 g/d)	1	−2.29	−3.47, −1.11	N/A	0.0001 *	N/A
Participants’ characteristic							
	AD	2	−1.22	−1.98, −0.46	57%	0.002 *	0.10
	AD with smoking	1	0.36	−0.55, 1.26	N/A	0.44	N/A
(12) Drug compliance							
Treatment duration (days)							
	<30	2	1.61	−1.61, 4.82	94%	0.33	<0.0001
	30–90	1	0.00	−0.74, 0.74	N/A	1.00	N/A
Alcohol consumption level risk							
	Medium (60–89 g/d)	1	0.00	−0.90, 0.90	N/A	1.00	N/A
	Low (30–59 g/d)	1	3.28	1.99, 4.58	N/A	<0.00001 *	N/A
	N/A	1	0.00	−0.74, 0.74	N/A	1.00	N/A
Participants’ characteristic							
	AD	1	3.28	1.99, 4.58	N/A	<0.00001 *	N/A
	AD with smoking	2	0.00	−0.57, 0.57	0.0%	1.00	1.00

Abbreviations: P^a^: *p*-value for heterogeneity; N/A: not available; OCDS: obsessive-compulsive drinking scale; PACS: Penn alcohol craving scale; AUQ: alcohol urge questionnaire; VAS: visual analog scale; * *p* ≤ 0.05.
